# Behavioral Changes in Aging but Not Young Mice after Neonatal Exposure to the Polybrominated Flame Retardant DecaBDE

**DOI:** 10.1289/ehp.11814

**Published:** 2009-06-17

**Authors:** Deborah C. Rice, W. Douglas Thompson, Elizabeth A. Reeve, Kristen D. Onos, Mina Assadollahzadeh, Vincent P. Markowski

**Affiliations:** 1 Environmental and Occupational Health Program, Maine Center for Disease Control and Prevention, Augusta, Maine, USA; 2 Maine Center for Toxicology and Environmental Health; 3 Department of Applied Medical Sciences and; 4 Department of Psychology, University of Southern Maine, Portland, Maine, USA

**Keywords:** behavior, behavioral effects, C57BL6 mouse, decabrominated diphenyl ether, fixed interval, fixed ratio, impulsivity, neonatal exposure, PBDE, perseveration, visual discrimination

## Abstract

**Background:**

After several decades of commercial use, the flame-retardant chemicals polybrominated diphenyl ethers (PBDEs) and their metabolites are pervasive environmental contaminants and are detected in the human body. Decabrominated diphenyl ether (decaBDE) is currently the only PBDE in production in the United States.

**Objectives:**

Little is known about the health effects of decaBDE. In the present study we examined the effects of neonatal decaBDE exposure on behavior in mice at two ages.

**Methods:**

Neonatal male and female C57BL6/J mice were exposed to a daily oral dose of 0, 6, or 20 mg/kg decaBDE from postnatal days 2 through 15. Two age groups were examined: a cohort that began training during young adulthood and an aging cohort of littermates that began training at 16 months of age. Both cohorts were tested on a series of operant procedures that included a fixed-ratio 1 schedule of reinforcement, a fixed-interval (FI) 2-min schedule, and a light–dark visual discrimination.

**Results:**

We observed minimal effects on the light–dark discrimination in the young cohort, with no effects on the other tasks. The performance of the aging cohort was significantly affected by decaBDE. On the FI schedule, decaBDE exposure increased the overall response rate. On the light–dark discrimination, older treated mice learned the task more slowly, made fewer errors on the first-response choice of a trial but more perseverative errors after an initial error, and had lower latencies to respond compared with controls. Effects were observed in both dose groups and sexes on various measures.

**Conclusions:**

These findings suggest that neonatal decaBDE exposure produces effects on behavioral tasks in older but not younger animals. The behavioral mechanisms responsible for the pattern of observed effects may include increased impulsivity, although further research is required.

Polybrominated diphenyl ethers (PBDEs) are a class of flame-retardant chemicals used in a variety of products, including textiles, plastics, and foam. PBDE levels have increased exponentially in the environment, wildlife, and human tissue since their introduction in the 1970s, with current doubling times of 2–5 years (Environmental Working Group 2003; Meironyté and Norén 1999; Rayne et al. 2003). Tissue levels of PBDEs in humans in the United States are 10–70 times higher than in Europe (Johnson-Restrepo et al. 2005; Schecter et al. 2003; Sjodin et al. 2008).

Two commercial mixtures, pentaBDE and octaBDE, are no longer produced in the United States or Europe. However, the fully substituted 2,2′,3,3′,4,4′,5,5′,6,6′-brominated diphenyl ether (decaBDE) continues to be widely used in the worldwide market. DecaBDE, like other PBDE congeners, is accumulated and bioconcentrated up the food chain (Burreau et al. 2004; Elliott et al. 2005; Jaspers et al. 2005, 2006; Vorkamp et al. 2005). It is readily absorbed after oral exposure (Mörck et al. 2003), crosses the placenta (Guvenius et al. 2003; Mazdai et al. 2003), and is excreted into breast milk (Guvenius et al. 2003; She 2005). DecaBDE is a dominant congener in house dust in the United States (Allen et al. 2007; Stapleton et al. 2005).

PBDEs may have effects on the nervous system similar to those of polychlorinated biphenyls (PCBs), a well-known class of developmental neurotoxicants in humans and animals. PBDEs and PCBs are structurally similar and produced similar effects on calcium homeostasis and second-messenger systems (Kodavanti and Derr-Yellin 2002; Kodavanti and Ward 2005; Kodavanti et al. 2005). Developmental exposure to either Aroclor 1254 or decaBDE disrupted a number of cell-signaling pathways in the hippocampus that are associated with cell proliferation, migration, axonal and dendritic extension, and synaptogenesis (Pruitt et al. 1999; Royland and Kodavanti 2008; Viberg et al. 2008). Developmental exposure to coplanar or *ortho*-substituted PCBs decreased the density of muscarinic acetylcholine receptors in the rat cerebellum, cerebral cortex, and hippocampus (Coccini et al. 2007; Eriksson 1988), whereas developmental PBDE exposure decreased hippocampal nicotinic receptors in the adult mouse (Fischer et al. 2008; Viberg et al. 2003a, 2004b) and muscarinic receptors in the rat hippocampus (Viberg et al. 2005).

Several studies have documented behavioral effects after developmental PBDE exposure. A single postnatal exposure to a number of individual congeners, including decaBDE, produced changes in locomotor activity in mice (Gee and Moser 2008; Viberg et al. 2003b, 2006b, 2007), cognitive effects in mice (Viberg et al. 2003a, 2006) or rats (Dufault et al. 2005), and changes in sexually dimorphic behavior in mice (Lilienthal et al. 2006). Apparently no human studies of the behavioral consequences of developmental exposure to PBDEs have been published. However, associations have been found between *in utero* PBDE exposure and cryptorchidism in newborn boys (Main et al. 2007) and decreased birth weight and length (Chao et al. 2006).

The possibility of delayed toxicity after early exposure has received increasing attention, with the recognition, for example, of the contribution of early environmental experience on adult obesity, hypertension, or endocrine response (Kajantie 2008; Ross et al. 2007; Simmons 2008; Victora et al. 2008). We previously reported changes in locomotor activity in young adult mice but not in their aging littermates after decaBDE exposure (Rice et al. 2007). In the present study, we tested mice during two developmental periods: in young adulthood and during aging (approximately 16 months of age).

In this study we used C57BL6/J mice as a developmental model. Previous studies were performed in the CD1 Swiss, NMRI, and C57BL6 mouse strains, with no indication of differential sensitivity to PBDE toxicity (Branchi et al. 2002, 2005; Gee and Moser 2008; Viberg et al. 2004a). In the present study, we used a repeated-dose regimen to correspond roughly to continuous exposure during the last trimester of human pregnancy, which represents a more environmentally relevant exposure paradigm than a single dose. However, this design allowed comparison with single-dose studies in which dosing occurred during the same overall postnatal period. In addition, approximately the same doses were used as in previous studies with decaBDE, although the total dose was higher in our studies.

We assessed the behavioral consequences of developmental exposure to decaBDE at both developmental periods on three behavioral tasks. Mice were first tested on a fixed-ratio (FR) schedule followed by a fixed-interval (FI) schedule of food reinforcement. These schedules have been used for decades in behavioral pharmacologic and toxicologic research, and the FI schedule is included in the U.S. Environmental Protection Agency (EPA) guidelines for neurotoxicity testing as an indicator of changes in nervous system function (U.S. EPA 1998). The two schedules generate different response patterns. The FR schedule generates a high rate of response, whereas the FI schedule generates a moderate response rate and also assesses temporal discrimination (timing). Although FR and FI are both simple operant schedules, apparently they have not been examined after developmental PBDE exposure. Developmental PCB exposure produced changes in FI performance in some studies (Holene et al. 1998; Lilienthal et al. 1990; Rice 1997) but not others (Holene et al. 1999; Rice and Hayward; 1998; Taylor et al. 2002). Differences may have been due to different congener mixtures, species differences, sex-specific effects, or other differences in dosing and testing protocol. Postnatal PCB exposure in monkeys produced differences in pause time on the FR across sessions (Rice 1997) with no other effects on FR performance. The FR in the present study was part of the training procedure before introduction of the FI. After the FI schedule, we assessed performance on a light–dark visual discrimination, a simple test of learning. Developmental PCB exposure produced deficits on visual discrimination tasks (Holene et al. 1995; Lilienthal and Winneke 1991; Rice and Hayward 1997), as did a commercial PBDE mixture (Dufault et al. 2005).

The subjects in the present study were littermates of mice in a study described previously (Rice et al. 2007). In that study, developmental exposure to decaBDE did not affect body weight, anogenital distance, crown–rump length, or physical development. Exposure-related changes were observed on locomotor activity and the ontogeny of some measures of neurologic function. There was also a dose-related reduction of total thyroxine in weanling animals.

## Materials and Methods

### Subjects and decaBDE exposure

Litters of C57BL6/J inbred mice (The Jackson Laboratory, Bar Harbor, ME) were culled to three female and three male pups on postnatal day (PND) 2. Litters were then assigned in a randomized fashion to a 0-, 6-, or 20-mg decaBDE/kg/day dose group. Each pup received a single daily oral dose from PND2 to PND15. The decaBDE was administered in a 1:10 egg lecithin:peanut oil mixture that was sonicated and hand-shaken to a 20% emulsion in sterile water (Eriksson et al. 2002). Fresh dosing solutions were prepared every other day and protected from light exposure. The decaBDE sample was generously donated by Å. Bergman, Department of Environmental Chemistry, Stockholm University. Its content was 99.5% decaBDE. Because neonatal mouse pups are too small to safely dose by intragastric gavage, the decaBDE emulsion was administered using a micropipette with 200-μL tips at a concentration of 5 μL/g body weight. Small amounts of dosing solution were placed in each pup’s mouth, and the micropipette tip was used to gently stimulate the perioral region to promote suckling and swallowing.

After weaning on PND21, offspring were housed with same-sex littermates until PND70. After PND70, offspring were housed individually and fed once daily, with the amount adjusted to control their body weights (males, 25–30g; females, 20–25g). Mice were fed standard pellet chow (Teklad Global 18% Protein Rodent Diet; Harlan, Indianapolis, IN). Housing rooms were on a 12-hr light/dark cycle in a barrier facility with an ambient temperature of 68 ± 2°F and 40–60% humidity. One male–female pair from each litter was assigned to begin behavioral training after reaching adulthood (mean ± SEM, 86.7 ± 3.5 days of age). A second male–female pair was assigned to begin training at about 16 months of age (mean ± SEM, 496.7 ± 10.7 days of age). The young cohort consisted of a total of 35 pairs from 11 control litters, 13 low-dose litters (6 mg/kg/day), and 11 high-dose litters (20 mg/kg/day). The aging cohort consisted of a total of 30 pairs from 9 control litters, 12 low-dose litters, and 9 high-dose litters. Because of age-related attrition in the aging cohort, 20 pairs of mice from 6 control, 7 low-dose, and 7 high-dose litters were available for the final visual discrimination procedure. Mice in the aging cohort were handled and weighed three times per week until testing began, and they experienced no other manipulations prior to behavioral testing. All animal procedures complied with approved institutional animal care protocols and were in accordance with National Institutes of Health guidelines (Institute of Laboratory Animal Resources 1996). Animals were treated humanely and with regard for alleviation of suffering. Animal care and welfare were supervised by a veterinarian and an American Association for Laboratory Animal Science–certified registered laboratory animal technologist.

### Behavioral apparatus

Subjects were tested in commercial operant chambers for mice (7 in. wide × 7 in. deep × 12 in. high; Coulbourn Instruments, Allentown, PA) controlled by Graphic State software, version. 3.01, for Windows XP. For lever-press training and the FR and FI procedures, a single response lever was positioned on the middle of one chamber wall and a feeder bin was centered on the opposite wall. For the visual discrimination procedure, the feeder bin was on one wall, and there were two levers on the opposite wall: one to the left of center and one to the right. A multicolored LED display positioned immediately above each lever served as the discriminative stimulus that a response on the associated lever would be reinforced. An overhead house light was on during sessions. Single food pellets (20 mg; Bio-Serv, Frenchtown, NJ) were automatically delivered into the feeder bin to reinforce correct responses.

### Lever press training and FR schedule

We used a continuous reinforcement schedule to train naïve mice to press the lever. During training sessions, the cue lights above the lever were illuminated; each press of the lever produced an audible click from the food dispenser, a 3-sec illumination of the food hopper, and delivery of a single 20-mg food pellet. Training sessions lasted for 6 hr or until a subject earned 60 food pellets. Subjects were considered to have learned the lever press response when they had completed a session with collection of 60 food pellets.

After subjects acquired the lever press response, training continued with 10 sessions under a FR1 reinforcement schedule in which one lever press resulted in delivery of one reinforcer. Each FR1 session lasted for 30 min or until a subject earned 60 food pellets. Sessions were 5 days/week. We examined the number of earned food pellets and the overall response rate.

### FI schedule

During the FI schedule, the cue lights above the lever were illuminated, and subjects were required to press the lever once after a 2-min interval had elapsed to receive a reinforcer. FI performance is typically characterized by an initial pause followed by an accelerating rate of response terminating in reinforcement. Each interval was separated by a 5-sec feed cycle period. The food hopper light was illuminated during the feed cycle, and the cue lights were extinguished. Feed cycle responses had no programmed consequences. Each session ended after 20 FIs or the first FI completed after 45 min had elapsed. Sessions were run once a day, 5 days/week, for a total of 60 sessions for both age cohorts.

We examined five dependent variables for FI performance: *a*)the overall response rate (the total number of responses divided by the session length); *b*)pause time (the average elapsed time before the first response of each 2-min interval); *c*)the run rate (number of responses per minute once the first response was made in any interval; i.e., not including the pause time); *d*)the index of curvature [IOC; a measure of the deviation from linearity of the response pattern (a greater IOC indicated better temporal discrimination)]; and *e*)the number of responses during the feed cycle.

### Visual discrimination procedure

The light–dark visual discrimination procedure began immediately after completion of the FI. The reinforced lever was programmed in a semirandomized, counterbalanced fashion so that each animal received the same number of trials on each lever within each session. At the beginning of each trial, the cue lights above the reinforced lever were illuminated and remained on until the subject made a response choice. The cue lights above the nonreinforced lever were dark. A correct response delivered a single food pellet, illuminated the food hopper light for 4 sec, and extinguished the cue lights. After the feed cycle, there was a 6-sec intertrial interval (ITI). An incorrect response produced a 2-sec timeout (TO) in which all lights in the chamber were extinguished. We used a correction procedure: An incorrect response resulted in the same lever being the correct one after the TO until a correct response was made. A trial included all choices until a correct response was made. Lever pressing during the ITI, TO, or feed cycle had no programmed consequences. Sessions were 5 days/week. All mice were tested for a total of 4 1sessions. Each session ended after 30 min or collection of 32 food pellets. By the eighth session, all mice were earning the 32 food pellets in the allotted 30 min.

For the young cohort, if a subject’s percentage of correct responding was at least 80% for two of three consecutive sessions, we implemented a discrimination reversal procedure in which the reinforced lever was the unlit rather than the lit lever. Because most of the mice in the young cohort did not achieve the criterion for the reversal procedure, the procedure was not used with the aging cohort.

The dependent variables were as follows: percentage correct (responding on the reinforced lever/total responding during the discrimination trials), the number of first-choice errors (the first incorrect response choice of a trial), number of perseverative errors (subsequent incorrect responses after the first incorrect response in a trial), number of TO plus ITI errors, total number of unreinforced responses (sum of all unreinforced responses including the first-choice errors, perseverative errors, TO, ITI, and food hopper responses); and the latency to a correct or incorrect response choice on each response choice. We also examined the number of sessions required to reach the criterion for the reversal procedure for the young cohort.

### Statistical methods

Before statistical analysis, the 60 total FI sessions were collapsed into 12 blocks, with each block representing the mean of five consecutive sessions. We examined the dependent variables from the FR, FI, and discrimination procedures using orthogonal polynomial regression analysis. Three parameters were calculated: the fitted mean, the linear slope, and the *y*-intercept for the linear term. We evaluated the *y*-intercept to determine if there were differences between exposure groups at the onset of each procedure. Significant differences in slope were interpreted as an indication of an exposure-by-session interaction. Parameter estimates for each end point were examined with analysis of variance (ANOVA) with SAS (GLM procedure; SAS Institute Inc., Cary NC). We always considered the litter to be the statistical unit of analysis, with sex as a within-litter factor and the decaBDE dose as a between-litter factor. We used the Huynh-Feldt adjustment to degrees of freedom when appropriate. Data were collapsed across sex if there was no sex-by-treatment interaction. If there was a sex-by-treatment interaction, males and females were analyzed separately.

In the young cohort, the number of sessions on the original discrimination completed by all subjects was 18; we analyzed these sessions by orthogonal polynomial analysis. The number of sessions to reach reversal criterion was compared by ANOVA. For mice that did not reach criterion by the 41st session, the number of sessions to criterion was considered 42. To get a better sense of discrimination learning in the young cohort, we assessed terminal performance by averaging the last three sessions completed on the original procedure for each subject and comparing by ANOVA. The mice that performed best and therefore reached criterion for reversal did not have the opportunity to perform the original procedure for 41 sessions; therefore, the differences in learning between individuals was presumably underestimated.

## Results

### Lever press training and FR performance

In the young cohort, 61 subjects acquired the lever press response in the first session, with the remaining mice reaching the criterion in the second (7 subjects) or third (2 subjects) session, with no exposure-related effects. All of the animals in the aging cohort acquired the lever press response in the first session.

For the FR1, we found no decaBDE exposure–related differences in the young cohort, although there was a main effect of sex on the mean number of earned food pellets [*F*(1,32)=5.23; *p=*0.03], with females earning more food pellets than males. The performance of the males and females was similar at the onset of the FR1, as reflected by a lack of effect on the *y*-intercept. The response rate of the females steadily increased, culminating in a greater number of earned food pellets by the end of the procedure. The divergence between the young males and females was also reflected by significant main effects of sex on the linear slope parameters for the number of earned food pellets [*F*(1,32)=7.41; *p=*0.01] and the response rate [*F*(1,32)=9.02; *p=*0.005].

**Figure 1:**
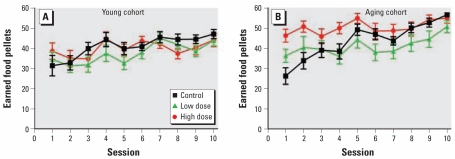
Number of earned food pellets (mean ± SE) among control and decaBDE-treated mice (low dose, 6 mg/kg; high dose, 20 mg/kg) across the 10 sessions of the FR1 procedure in the young cohort (*A*) and the aging cohort (*B*). Male and female data were averaged within each litter.

In the aging cohort, there was a significant main effect of decaBDE exposure on the slope parameter for the number of earned food pellets (Figure 1) [*F*(2,23)=4.29; *p=*0.03]. Posthoc analyses indicated that the slopes for the low- and high-dose groups were lower than for the control group (*p=*0.05 and *p=*0.01, respectively). The control group earned the fewest reinforcers at the beginning of the task, and the high-dose group earned the most; by the end of the 10 sessions, all dose groups earned about the same number of reinforcers. There were no other effects in the aging cohort.

The differences observed in the old cohort but not the young were the result of differences in performance in both the control and decaBDE high-dose groups. The number of reinforcers earned by the controls at the start of the assessment was greater in the young cohort, with little change over the course of the 10 sessions. In contrast, the number of reinforcers earned by the controls in the older cohort was lower than in the young cohort, but it increased across sessions to a higher number by the end of the 10 sessions. The aging high-dose group earned more reinforcers across the procedure than groups in the young cohort, whereas the performances of the low-dose aging and young cohorts were similar.

### FI performance

We found no evidence of exposure-related effects on FI performance in the young cohort, but we did observe a sex difference for the postreinforcement pause time at the beginning of the experiment, as indicated by the *y*-intercept [*F*(1,32)=6.85; *p=*0.01]. Females had a shorter pause time during early sessions, with the sexes being comparable by the eighth session block. In both males and females, the pause time lengthened over the course of the procedure. The number of feed cycle responses was very low and did not vary according to exposure, sex, or session block.

**Figure 2:**
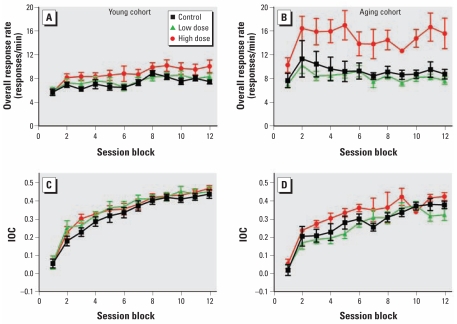
FI overall response rate (*A, B*) and IOC(*C,D*) among control and decaBDE-treated mice (low dose, 6 mg/kg; high dose, 20 mg/kg) across the 60 sessions of the FI procedure in the young cohort (*A,C*) and aging cohort(*B,D*). Values are mean ± SE. Each session block is the mean of five consecutive sessions; male and female data were averaged within each litter.

In contrast, we did observe treatment-related effects on FI performance in the aging cohort. There were significant main effects of decaBDE exposure on the fitted mean for the overall response rate (Figure 2) [*F*(2,23)=7.97; *p=*0.002], with the high-dose group having a marginally higher response rate than the control group [*F*(1,13)=4.32; *p=*0.06]. There was also a significant main effect of exposure on the IOC fitted mean [*F*(2,23)=4.13; *p=*0.03], with the low-dose group having a marginally lower IOC than controls [*F*(1,17)=3.90, *p=*0.06]. The main effect was due largely to differences between the high- and low-dose groups rather than differences between either of the treated groups and controls. We observed a significant main effect of decaBDE exposure on the slope for the overall response rate [*F*(2,23)=4.82; *p=*0.02], although posthoc comparisons did not reveal significant differences. The run rate was not affected by exposure, but there was a significant main effect of sex on the fitted mean [*F*(1,21)=6.19; *p=*0.02], with males responding at a faster rate than females. We saw no effects of exposure on pause time or on *y*-intercept parameters.

Comparing FI performance for the young and aging cohorts, we found the differential effect on response rate was the result of differences in the performance of the treated aging animals, with the high-dose aging group having a higher response rate than the other five dose groups. For the IOC, the aging controls exhibited poorer temporal discrimination than the young controls, with the performance of the treated groups showing no consistent pattern of difference from control.

### Visual discrimination performance

Subjects in the young cohort began to reach the reversal criterion after 18 sessions of the visual discrimination procedure. Polynomial regression analysis over the first 18 sessions did not reveal any exposure-related effects. DecaBDE exposure did not affect the number of sessions to reach criterion, although there was a significant main effect of sex [*F*(1,32)=12.42; *p=*0.001], with females requiring fewer sessions than males. The mean latencies for both reinforced and unreinforced responses varied little over the course of the procedure and were not affected by exposure.

For terminal (asymptotic) performance, the decaBDE exposed groups in the young cohort emitted more nonreinforced responses in every category (Table 1). We observed a significant main effect of decaBDE exposure on total unreinforced responses [*F*(2,32)=3.27; *p=*0.05], with the high-dose group making significantly more errors than controls. There were main effects of sex on first-choice errors [*F*(1,32)=16.70; *p=*0.0003], percentage correct [*F*(1,32)=10.22; *p=*0.003], total unreinforced responses [*F*(1,32)=6.21; *p=*0.02], TO responses [*F*(1,32)=6.32; *p=*0.02], and perseverative errors [*F*(1,32)=4.56; *p=*0.04]. In each case, males made more errors than females. decaBDE exposure did not affect the mean asymptotic response latencies for reinforced or unreinforced responses.

**Figure 3:**
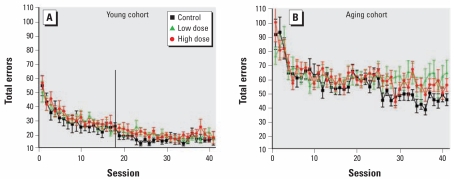
Total number of errors (mean ± SE) among control and decaBDE-treated mice (low dose, 6 mg/kg; high dose, 20 mg/kg) across 41 sessions of the visual discrimination procedure in the young (*A*) and aging (*B*) cohorts. Male and female data were averaged within each litter. In the young cohort, the vertical line at the 18th session indicates the last session that included all of the subjects. After session 18, young subjects began to reach the criterion for the reversal procedure.

We found a number of significant effects of decaBDE exposure on visual discrimination in the aging cohort (Table 2). There was a main effect of exposure in the aging cohort for the total percentage correct responses for the linear slope [*F*(2,17)=5.25; *p=*0.02], with the slope of the high-dose group being less than that of controls [0.39 vs. 0.74; *p=*0.02] (Figure 3). The error rate of the control group decreased over sessions, and the overall error rate in the high-dose group decreased more slowly than controls. This was the result of a higher number of total errors over the last 15 or so sessions in the high-dose group.

**Figure 4:**
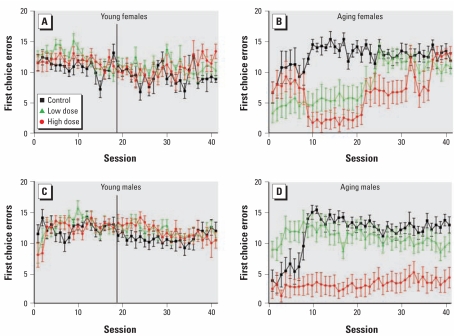
Number of first-choice errors (mean ± SE) among control and decaBDE-treated mice (low dose, 6 mg/kg; high dose, 20 mg/kg) in the visual discrimination procedure in the young(*A, C*) and aging(*B, D*) cohorts by sex. Because there was an exposure-by-sex interaction, females and males were analyzed separately. In the young cohort data plots, the vertical bar at the 18th session marks the last session that included all of the subjects.

decaBDE-treated mice made fewer first-choice errors than controls (Figure 4), with the pattern differing somewhat between females and males. There was a main effect of exposure on the fitted mean number of first-choice errors [*F*(2,17)=58.55; *p <*0.0001], as well as a significant exposure-by-sex interaction [*F*(2,17)=10.18; *p=*0.0012]. Posthoc comparisons revealed that the high-dose females, low-dose females, and the high-dose males made fewer first-choice errors than their same-sex controls (*p<*0.0001 for each). We observed an exposure-by-sex interaction for the linear slope parameter [*F*(2,17)=4.59; *p=*0.03]. The low- and high-dose males changed at different rates than controls over the course of the procedure (*p=*0.005 and 0.04, respectively). The slope parameter estimates for males in both exposed groups were lower than that of the male control group, indicating a shallower slope. For *y*-intercept, significant main effects of exposure [*F*(2,17)=9.70; *p=*0.002] indicated that performance differed between groups at the beginning of the experiment. An exposure-by-sex interaction for the *y*-intercept [*F*(2,17)=11.41; *p=*0.0007], with the low-dose females and the high-dose male and female *y*-intercepts significantly lower than intercepts for the same-sex controls, indicated fewer first-choice errors at the beginning of the task (*p=*0.007, *p=*0.01, and *p <*0.0001, respectively).

**Figure 5:**
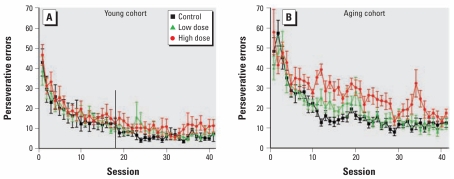
Number of perseverative errors (mean ± SE) among control and decaBDE-treated mice (low dose, 6 mg/kg; high dose, 20 mg/kg) across the visual discrimination procedure in the young (*A*) and aging (*B*) cohorts. Male and female data were averaged within each litter. In the young cohort data plot, the vertical line at the 18th session marks the last session that included all of the subjects.

In contrast to the better performance of the treated groups on the initial response of a trial, decaBDE-treated mice in the aging cohort made more perseverative errors than controls after an initial error (Figure 5). There was a main effect of exposure [*F*(2,17)=4.20, *p=*0.03], with the high-dose group making more perseverative errors than controls (*p=*0.03). A significant main effect of exposure on the slope parameter [*F*(2,17)=4.22; *p=*0.03], with the slope of the high-dose group being marginally shallower than the control or the low-dose group, indicated slower improvement in performance (*p=*0.09). There was also an exposure-by-sex interaction for the slope [*F*(2,17)=8.58; *p=*0.003]. The number of errors made by the low-dose females and the high-dose males declined more slowly than for their same-sex controls across sessions (*p=*0.03 and *p=*0.05, respectively). In addition, an exposure-by-sex interaction for the *y*-intercept [*F*(2,17)=5.01, *p=*0.02] indicated a difference between groups at the beginning of the experiment for the fitted curves, but there were no significant differences on posthoc analysis.

**Figure 6:**
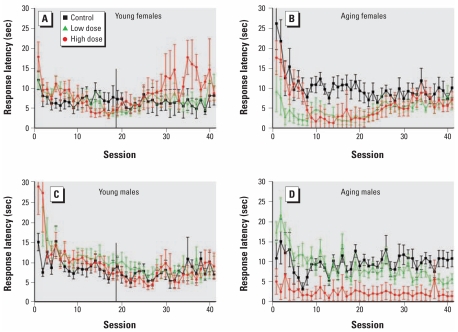
Latency to respond (mean ± SE) among control and decaBDE-treated mice (low dose, 6 mg/kg; high dose, 20 mg/kg) across the visual discrimination procedure in the young (*A,C*) and aging(*B,D*) cohorts by sex. Because there was an exposure-by-sex interaction, females and males were analyzed separately. In the young cohort (*A,C*), the vertical line at the 18th session marks the last session that included all of the subjects.

Older exposed animals also responded more quickly than controls when making a choice at the beginning of a trial (Figure 6). We observed a main effect of exposure [*F*(2,17)=23.16; *p <*0.0001] and an exposure-by-sex interaction [*F*(2,17)=4.01; *p=*0.04] for the mean response latency (low-dose females vs. control, *p=*0.02; high-dose females vs. control, *p=*0.003; high-dose males vs. control, *p <*0.0001). There was also an exposure-by-sex interaction for the slope parameter [*F*(2,17)=5.12; *p=*0.02]. The latencies for the control and high-dose males did not change much across sessions after the first few sessions, whereas the latencies for low-dose males increased across sessions (*p=*0.03 vs. control). The response latency for the control females was stable after the first few sessions, whereas the latencies of the treated groups decreased and then increased (*p=*0.02 for low-dose vs. control). We also observed an effect on the *y*-intercept for response latency [*F*(2,17)=10.57; *p=*0.001], as well as an exposure-by-sex interaction [*F*(2,17)=5.93, *p=*0.01]. The fitted intercepts of the low-dose female and the high-dose female and males were significantly lower than the same sex controls, indicating lower latencies at the start of the task (*p=*0.01, *p=*0.03, and *p=*0.0009, respectively).

The only other significant finding was a main effect of sex on the sum of all other errors [*F*(1,17)=8.13; *p=*0.01], with females making more errors than males.

Comparison of the young and aging cohorts revealed differences in control performance, as well as performance of the treated groups compared with controls. For total errors, all groups in the young cohort performed better than those in the aging cohort, with only the higher-dose older cohort being different from their controls. The aging control groups of both sexes initially made fewer first-choice errors than their younger controls, which increased over the first 10 or so sessions to stable performance with more errors than their young littermates. In contrast, the aging decaBDE-treated animals made fewer errors than young animals or the aging controls. For perseverative errors, the aging control group performed more poorly than their young counterparts, particularly during early sessions; the performance of the high-dose aging mice was worse than that of any other group. For response latency, aging controls of both sexes appeared to respond more slowly than their young littermates. decaBDE-treated mice in the aging cohort in general had shorter latencies than aging controls or young animals, which were particularly marked in the high-dose males.

## Discussion

An important aspect of the present study is the finding of effects in aging mice that were not present in their young littermates. The issue of delayed neurotoxicity has received a fair amount of attention (e.g., Weiss 2000a, 2000b). This is perhaps best documented for methylmercury, which produced delayed neurotoxicity on numerous outcomes in both animals and humans (Newland et al. 2004; Newland and Rasmussen 2000; Rice 1996). Viberg et al. (2003a, 2004a) found that neonatal exposure to several different PBDE congeners impaired the normal motor habituation as animals became familiar with a novel test environment. These authors reported that the effects of PBDE congeners 99 or 153 on habituation were significantly more severe in mice that were ≥ 6 months of age compared with younger animals (Viberg et al. 2003a; 2004a). The same effect was observed in animals exposed to decaBDE, although it was less striking (Viberg et al. 2003b). In general, few studies have compared performance in young and aging animals after perinatal exposure, yet it is of critical importance to the understanding of the health implications of exposure to environmental chemicals.

In the present study, we found no effects of decaBDE exposure on FI performance in young adult C57BL6/J mice. However, older littermates exposed to high-dose decaBDE (20 mg/kg/day) performed less efficiently than corresponding controls, emitting twice as many responses for the same number of reinforcers. FI performance has not previously been assessed after exposure to any of the PBDEs, but the effects of developmental PCB exposure on FI performance have. A robust increase in response rate was observed in juvenile monkeys dosed postnatally with a PCB mixture representative of human breast milk (Rice 1997). In rats, developmental exposure to various PCBs produced increased rates of FI responding in some studies (Holene et al. 1998; Lilienthal et al. 1990). In these rat studies, PCBs also produced increased motor activity, which was observed in young but not aging mice in our previous study (Rice et al. 2007). Other studies in rats found no effect on FI performance (Holene et al. 1999; Taylor et al. 2002).

The higher response rate of the high-dose aging mice may represent increased impulsivity. The PCB-exposed monkeys discussed above also displayed increased response rates and a concomitant decrease in the number of reinforced responses on a differential reinforcement of low rate (DRL) schedule, which punishes failure to inhibit inappropriate responding (Rice 1998). Stewart et al. (2006) observed the same pattern of failure of response inhibition on the DRL schedule in children as a function of PCB exposure; these children also exhibited failure of response inhibition on a vigilance task, thereby making more errors (Stewart et al. 2003, 2005). Developmental lead exposure also reliably produced an increase in response rate on the FI, as well as increased impulsivity on a variety of tasks (Rice 2006). Indeed, a higher FI response rate predicted failure of response inhibition in individual children on a test specifically designed to assess planning strategy (Darcheville et al. 1992, 1993), and children with attention deficit hyperactivity disorder (ADHD) responded at a higher rate on the FI than non-ADHD children (Sagvolden et al. 1998).

In the present study, all dose groups in both cohorts acquired the typical scalloped-shaped response distribution typically generated by the FI schedule, as indicated by increasing IOCs across sessions, and there was no consistent difference in performance for the dosed groups compared with controls on temporal discrimination. (The IOC was higher in the high-dose decaBDE group than in controls but lower in the low-dose group.) In studies in monkeys (Rice 1997) and rats (Lilienthal et al. 1990) exposed to PCBs, there was some evidence that treated animals made more responses early in the interval, concomitant with an overall increase in response rate, but there was no evidence of changes in the overall temporal patterning of responses. Similarly, environmental pollutants such as lead or methylmercury typically have shown little or no effect on temporal discrimination on the FI (Rice 1992, 2006).

Performance on the FR1 schedule in the present study was consistent with that on the FI, in that there was no effect in the young animals, whereas the high-dose older animals emitted more responses during the first few sessions of the FR schedule. It is tempting to speculate that the increased response rate on the FI schedule may be a consequence of the behavioral history of performance on the FR1 schedule, with the treated mice failing to respond to new contingencies. This explanation is not compelling, however, because all three dose groups were performing similarly at the end of the 10 FR1 sessions, and the response rate on the first block of FI sessions did not differ among groups. We used the FR primarily to provide the mice with a history of lever-pressing in preparation for the FI.

We observed several effects in the aging cohort on the visual discrimination task. Aging high-dose decaBDE mice decreased their total error rate more slowly over the course of the experiment than their controls, which was the result of a higher error rate during the last 15 or so sessions. This finding is consistent with that of increased total nonreinforced responses during the terminal sessions in the young cohort, which was the only effect observed in the young cohort. The types of errors also were different between control and treated groups in the aging cohort. Exposed mice exhibited a greater degree of accuracy on the first response of a trial (fewer first-choice errors) than did their controls, but they emitted more perseverative errors (repeated errors on the same incorrect lever after an initial incorrect response).

It is difficult to explain the error patterns based only on results of the present study. However, the fewer number of first-choice errors exhibited by the decaBDE-treated groups should not be interpreted as necessarily indicating a salutary effect of decaBDE. In other studies, lesions in various parts of the brain have also resulted in improved performance in several tasks in monkeys (Mahut 1971, 1972; Zola and Mahut 1973) and rats (Tenas-Huerga et al. 1998; White and McDonald 1993). In addition, better performance on learning tasks in some circumstances, as a result of developmental exposure to PCBs or dioxins, has been observed in both rats (Schantz et al. 1996; Seo et al. 1999; Widholm et al. 2003) and monkeys (Bowman et al. 1990; Schantz et al. 1989). Facilitated performance on a visual discrimination task and impaired performance on a spatial discrimination task were observed in male 2,3,7,8-tetrachlorodibenzo-*p*-dioxin (TCDD)-exposed rats tested in an operant chamber (Seo et al. 1999; Widholm et al. 2003). The authors postulated that the TCDD-exposed rats had an increased tendency to explore, which would result in poorer performance in the spatial task (in which animals were rewarded for confining responses to the same lever), but might result in better performance on the visual task (in which changing response levers was required). This would be consistent with results in the present study for the decrease in first-choice errors, because the mice were required to switch levers on some trials. However, it would not explain the increased perseverative errors. In the above studies, first-choice and perseverative errors were not analyzed separately during the acquisition of the task. In a study of the effects of *inutero* PCB exposure in monkeys (Schantz et al. 1989), exposed animals exhibited better performance on a shape discrimination problem after testing on a spatial discrimination problem with irrelevant shape cues. The authors suggested a failure to learn the irrelevance of the shape cues as a possible explanation for the better performance. This would not explain the present findings because there were no irrelevant cues. In a study in lead-exposed rats, young and old animals, but not young adults, performed better than controls on a delayed spatial alternation task after extensive exposure to a cued alternation training task (Cory-Slechta et al. 1991). These authors postulated perseveration of the learned motor pattern in lead-treated rats as an explanation for the better performance. This explanation seems reasonable because it is well established that lead exposure produces perseverative behavior in both children and animals (Rice 2006).

In the present study, the correct lever was signaled by cue lights above it, with the incorrect lever unlit. This followed extensive testing on the FR and FI schedules, which used only one lever with cue lights lit above it when the schedule was in effect. It may be that the lower first-choice error rate of the treated mice was the result of a type of perseveration on the familiar (lit) lever and failure to explore the unlit one. For the aging control group, an initial perseveration on the lit lever, and consequently very low error rate, was followed by exploration of the unlit lever, resulting in an increase in first-choice errors over the first several sessions followed by an error rate that decreased slightly across subsequent sessions. The performance of the low-dose decaBDE males was not very different from that of controls after the first 10 sessions, whereas the high-dose decaBDE males continued to respond on the lit lever, with very few first-choice errors throughout the task. The female decaBDE-treated groups exhibited fewer initial errors during most of the task but reached control levels by the end of testing. The very low first-choice error rate at the beginning of the task in all groups, followed by an increase in errors, suggests perseveration on the lit lever rather than simply positive transfer for responding on the lit lever. Control animals did not persist in this pattern, whereas some treated groups did. The higher rate of perseverative errors in the high-dose males would be a further manifestation of the same behavioral deficit; once a lever was chosen, the mouse continued to respond on it. Sustained perseverative responding over many sessions has also been observed after lead exposure (Rice and Karpinski 1988); therefore, this hypothesis is not untenable.

Perseveration has not been studied previously with respect to PBDEs, and has been less well studied with respect to PCB exposure than for lead. Monkeys exposed to a PCB mixture representative of human breast milk exhibited more perseverative errors on a spatial delayed alternation task (Rice 2000), with little evidence for perseveration on tasks that produced perseverative behavior in lead-exposed monkeys in the same laboratory. Perseveration represents an inability to respond appropriately to the consequences of previous choices (learn from past mistakes). Indeed, it is tempting to speculate that perseveration represents an aspect of impulsivity (inability to inhibit inappropriate or unrewarding responding). In the present study, some of the older decaBDE-treated groups had a shorter latency to respond on the visual discrimination task than controls, consistent with the higher response rate of treated mice on FI performance. This may represent another manifestation of increased impulsivity in the treated groups. This explanation is also consistent with the results of light–dark discrimination in the young cohort, in which we observed an increase in overall nonreinforced responding, suggesting increased impulsivity. It may be that the aging cohort actually exhibited increased impulsivity compared with the young cohort, which was manifested largely as perseverative behavior. However, the hypothesis that increased impulsivity underlies the effects observed in the present study requires considerable further testing on tasks that measure specific behavioral domains. Because other studies also found an age-related increase in perseveration in the C57BL6/J mouse (Dean et al. 1981), it could be argued that decaBDE exposure accelerated a typical age-related process.

Performance on cognitive tasks after developmental PBDE exposure has been assessed in several studies. Rats exposed neonatally to the commercial pentaBDE mixture DE-71 committed more errors during a visual discrimination task and required more sessions to reach an acquisition criterion but were not impaired on a test of attention (Dufault et al. 2005). The Morris Water Maze employs negative reinforcement (escape from a water bath onto a platform) to evaluate learning and spatial memory; a single dose of BDE-99 had no effect on the amount of time required to find the escape platform during initial acquisition, but treated mice took significantly longer to find the platform when it was relocated to a different quadrant (Eriksson et al. 2001), suggesting perseveration for the previous position. Mice exposed to the octaBDE congener 203 were also impaired on the second but not the first phase of the task (Viberg et al. 2006), whereas exposure to the hexaBDE congener 153 produced deficits in both original learning and learning the new position (Viberg et al. 2003a).

In summary, we found that postnatal exposure to decaBDE produced changes in performance on behavioral tasks when mice were tested during aging, with minimal evidence of impairment during young adulthood. The findings of the present study extend our previous report of changes in motor activity, delayed sensorimotor development, and decreased thyroid hormone in littermates of the mice reported here (Rice et al. 2007). The underlying behavioral mechanisms of the effects observed in the aging mice in the present study remain to be elucidated, although increased impulsivity may explain at least some of the observed effects.

## Correction

In Figures 3A and 5A of the original manuscript published online, the vertical line should have indicated the 18th session. The figures have been corrected here.

## Figures and Tables

**Table 1:** Unreinforced responses (mean ±SE) among control and decaBDE-treated mice (low dose, 6 mg/kg; high dose, 20 mg/kg) in the young cohort averaged across the last three sessions of visual discrimination.

	Incorrect first- choice errors	Perseverative errors	TO and ITI responses	Total nonreinforced responses
Control	8.1 ± 0.7	4.4 ± 0.7	17.4 ± 1.3	32.2 ± 2.2
Low dose	10.2 ± 0.6	6.1 ± 0.9	21.3 ± 3.3	41.2 ± 3.2
High dose	9.0 ± 0.7	7.3 ± 1.5	24.1 ± 3.1	44.3 ± 4.4*
Male	10.5 ± 0.5	7.1 ± 0.8	22.8 ± 3.1	44.1 ± 3.3
Female	7.8 ± 0.5	4.7 ± 0.8	19.1 ± 1.4	34.5 ± 2.2

* *p <*0.05 compared with the control group.

**Table 2:** Summary of significant differences among decaBDE-treated mice (low dose, 6 mg/kg; high dose, 20 mg/kg) compared with controls on visual discrimination performance in the aging cohort.

	Mean		Slope		*y*-Intercept	
	Main effect of exposure	Exposure-by-sex interaction	Main effect of exposure	Exposure-by-sex interaction	Main effect of exposure	Exposure-by-sex interaction
Percent correct			High dose*			

Perseverative errors	High dose*		High dose*	Low-dose female,*		
				high-dose male*		

First-choice errors	Low dose,**	Low-dose female,**		Low-dose male,*	High dose**	Low-dose female,*
	high dose**	high-dose female,**		high-dose male*		high-dose female,*
		high-dose male**				high-dose male**

Response latency	Low dose,**	Low-dose female,*		Low-dose male,*	Low dose,*	Low-dose female,*
	high dose**	high-dose female,*		low-dose female*	high dose**	high-dose female,*
		high-dose male*				high-dose male*

* p ≤ 0.05.

** p ≤ 0.001.
